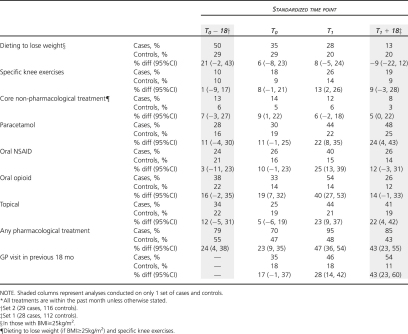# When Knee Pain Becomes Severe: A Nested Case-Control Analysis in Community-Dwelling Older Adults

**DOI:** 10.1016/j.jpain.2009.01.323

**Published:** 2009-08

**Authors:** George Peat, Elaine Thomas

**Affiliations:** Arthritis Research Campaign, National Primary Care Centre, Keele University, Keele, United Kingdom

**Keywords:** Knee pain, coping, osteoarthritis, Knee Clinical Assessment Study

## Abstract

Mild knee pain is a common symptom in later life. Despite this fact, there are few data on the impact of it worsening or how individuals alter their appraisals and behavior when it becomes severe. We sought to describe the changes that accompany a substantial deterioration in characteristic knee pain. A nested case-control analysis of existing cohort data identified 57 adults aged over 50 years experiencing progression from mild to severe characteristic pain intensity 18 months later and compared them, before and after this transition, with 228 controls whose knee pain did not progress. Worsening knee pain was accompanied by a marked increase in pain frequency and extent, functional limitation, depressive symptoms, catastrophising, praying and hoping, and use of oral and topical analgesia. Most individuals consulted a general practitioner either during or after this episode. Although relatively rare, substantial deterioration in knee pain has a major impact on those affected. Timely presentation to primary care, addressing potentially unhelpful appraisals and coping strategies, reinforcing core nonpharmacological management, and future research to identify triggering events for substantial deterioration and loss of adequate pain control should be part of an agenda to improve care for this important minority of older adults with knee pain.

**Perspective:**

This article describes what happens when the common symptom of mild knee pain in later life becomes significantly worse. The results may help clinicians understand the health impact, changes in patient appraisal and coping, and treatments that typically accompany this change in symptoms.

Knee pain is the most common pain complaint presented to the general practitioner by older adults.^29^ Yet, as is true of pain in general and many other symptoms, those with knee pain visiting the doctor represent only the tip of a much larger “iceberg of morbidity” in the general population.^14,16,27,48,57^ One recent study in the United Kingdom found that only 19% of older adults who reported knee pain in a health survey had a record of consulting their GP for this problem in the following 18 months.^22^ In part, this reflects the fact that in the general population the majority of knee pain is likely to be relatively mild in nature^20^. Many people do not regard such symptoms as illness but as a sign of “normal aging”.^32,48,62^

Early work identified the importance of self-treatment and “no-action decisions”^40^ and subsequent surveys have identified a wide repertoire of techniques used by older adults to cope with and control their pain.^4,8,37,54^ These include the use of prescription and over-the-counter (OTC) analgesia and a range of cognitive- and behavioral-coping strategies. What determines the choice of approach by an individual is not fully known but the use of most of these modalities appears to be closely related to pain intensity.^3,41^ Cross-sectional studies, including older adults with pain or osteoarthritis (OA) (the most common diagnosis of knee pain in older people), describe a higher proportion of health-care consultations,^3,5^ prescription analgesia,^30,45^ frequent analgesia,^55^ alternative care and self care,^3^ catastrophising, and praying and hoping^41^ among those people reporting more severe pain. Longitudinal studies have suggested that the coping strategies adopted by patients may predict future levels of pain or disability although the precise findings from these studies are conflicting. In a secondary analysis of clinical trial data in patients with knee OA, Steultjens et al^47^ found that the use of resting (a passive strategy) and pain transformation (an active strategy) were associated with more poorly-observed functional performance and higher pain, respectively. An observational study of adults with knee pain presenting to general practice using the same measures of coping failed to replicate these findings for self-reported outcomes at 3 and 12 months.^56^ In their original study of 82 community-dwelling adults with OA, Hampson et al^15^ hypothesized that passive coping strategies were the result of an appraisal of pain as more severe and serious but found no association between intensity appraisal and active coping. Together these studies provide a useful description of the association between current or future pain intensity and cognitive and behavioral coping measured at 1 point in time. However, joint pain in later life is typically variable over time,^10^ and these studies do not directly address the question of whether individuals alter their coping behavior when pain becomes more severe. To do this, we need repeated measures of both pain intensity and also of coping strategies.

To investigate this question, we conducted nested case-control analyses using data from a 3-year prospective, population-based observational cohort study of older adults with knee pain. We were interested in a particular transition: The change from mild to severe characteristic pain. Our hypothesis was that substantial worsening of knee pain would trigger the higher use of all coping strategies, pharmacological and nonpharmacological treatments, and general practice consultation compared with controls. Furthermore, prior to substantial worsening, when both cases and controls had comparable levels of mild knee pain, no differences in the above variables would be observed between cases and controls.

## Methods

We conducted a nested case-control analysis, sampling cases and controls from an existing population-based prospective observational cohort study; the Clinical Assessment Study of the Knee, abbreviated as CAS(K).

### Source of Cases and Controls

The CAS(K) cohort comprises 819 individuals with knee pain, aged 50 years and over, registered with 3 general practices (irrespective of their actual consultation pattern). Cohort members were recruited between August 2002 and September 2003 from a 2-phase postal survey. Respondents providing written informed consent to further contact attended a research clinic that included a standardized clinical interview, examination, and plain radiographs. Participants were sent a postal follow-up questionnaire 18 months (778 responded, crude response 95%) and 3 years (697 returned, crude response 85.1%) after study entry. All participants provided written informed consent to take part in the study. 742 (90.6%) participants provided additional written informed consent specifically to review their general practice medical records. The study was approved by North Staffordshire Local Research Ethics Committee. Full details of the study design and methods have been previously presented.^35,36^

### Data Collection

Information was gathered by self-complete questionnaires at study entry, 18-month follow-up, and 3-year follow-up.

### Pain and General Health Characteristics

Measures that were repeated at each time point included: Characteristic pain intensity measured using items from the Chronic Pain Grade^58^ (CPG). Characteristic pain intensity was calculated as the mean of 3 11-point numerical rating scales (NRS), for current pain intensity, average pain intensity in the past 6 months, and worst pain intensity in the past 6 months, multiplied by 10. Scores range from 0 (least pain) to 100 (most pain); Frequency of knee pain recorded as the number of pain days in the previous 6 months (0, 1–30, 31–89, 90+)^58^ and was analysed as persistent pain^59^ (90+ days) vs nonpersistent pain (0–89 days); Pain extent measured using a whole-body manikin on which respondents shaded all areas of pain experienced in the previous month. This was coded using a predefined template and scored as the number of mutually-exclusive areas with shading (range: 0–44);^28^ Night pain measured used a single item from the Western Ontario and McMaster Universities Osteoarthritis Index (WOMAC) Likert version 3.0 Pain subscale that asks about the severity of pain “at night while lying in bed” (none, mild, moderate, severe, extreme)^6^ and was dichotomized to severe/extreme vs none/mild/moderate); Physical function measured by the Physical Functioning scale of the WOMAC^6^ (WOMAC-PF). Scores can range from 0 (least functional limitation) to 68 (most); Anxiety and depression using the Hospital Anxiety and Depression Scale^64^ (HADS). This provides 2 scores, 1 for anxiety and 1 for depressive symptomatology, each ranging from 0 (least) to 21 (most). The HADS is a commonly used and extensively-tested measure with adequate psychometric properties in the general population;^7^ Self-rated health using a single item from the SF-12^61^ was used (“In general, would you say your health is”). Responses were dichotomized into excellent/very good/good vs fair/poor.

### Coping and Appraisal, and Treatment

Coping and appraisal was gathered using the 1-item Coping Strategies Questionnaire^18^ (CSQ). This shortened version of the original CSQ^43^ was developed to enable the inclusion of brief indicators of coping strategies in epidemiological studies. It comprises 1 item each for the subscales on Distraction, Reinterpreting Pain Sensations, Catastrophising, Ignoring Sensations, Praying and Hoping, Coping Self-Statements, and Increased Behavioural Activities, each recorded on a 7-point scale, ranging from 0 (“never do”) to 6 (“always do”). In this population sample, responses were strongly positively-skewed. The proportion of respondents reporting “never do” ranged from 26% (Coping Self-Statements) to 67% (Praying and Hoping). Responses for these analyses were therefore simply dichotomized as 0 (“absent”) or 1–6 (“present”). The 1-item CSQ was administered at 18-month and 3-year follow-up but not at study entry; Treatment was gathered using a modified version of the KNEST^19^ and a checklist of treatment options for the knee that participants had used in the previous month. These covered dieting to lose weight, doing specific knee exercises (eg quadriceps strengthening), taking oral nonsteroidal anti-inflammatory drugs (NSAID), oral weak opioids (eg dihydrocodeine) and topical creams or gels (including topical NSAIDs and rubefacients). These are among the most commonly-recommended treatments for knee OA.^21,33,42,63^ No distinction was made in this study on the source of analgesia (ie prescription vs OTC).

To identify all knee-related GP consultations, a review of the general practice consultation records from baseline to 3 years was undertaken for all participants who specifically provided written informed consent to accessing their medical records. Doctors at the practices routinely code and enter details of all patient consultations on computer. Individual problems are coded separately during each consultation. The participating practices are members of the Keele GP Research Partnership and the completeness of their coding of consultations is subject to annual quality review.^38^ All consultations related to the knee were identified through a search of the Read code (formal diagnostic and symptom codes) and free-text entries (full details of the search strategy are available from the authors). Free-text entries were independently assessed by 2 researchers. Disagreements were resolved by consensus between the 2 raters.

### Defining ‘Substantial Deterioration’ in Characteristic Knee-Pain Intensity

We were interested in the transition from mild pain to severe pain and attempted to omit very short-term fluctuations in pain intensity. Substantial deterioration of knee pain was therefore defined as a change in characteristic pain intensity from <50/100 (‘mild’) to ≥70/100 (‘severe’) between 2 consecutive reports 18 months apart. The cut-offs for characteristic pain intensity were selected to be consistent with those suggested for 11-point pain numerical-rating scales (<5 = mild, 5 to <7 = moderate, 7–10 = severe^34^). This definition of substantial deterioration would generally imply at least a 50% increase in characteristic pain intensity over an 18-month period.

### Selection of Cases and Controls

Cases were defined as participants who had experienced a substantial deterioration in characteristic knee-pain intensity. After 3 years of follow-up we had 2 sets of such cases “nested” in our cohort population: a first (Set 1) who had mild knee pain at study entry which had become severe at 18-month follow-up and a second (Set 2) who had mild knee pain at 18-month follow-up which had become severe by 36-month follow-up. These 2 groups together formed the total “case” population for this analysis.

We identified a control group from the cohort population for each case set (control: cases = 4:1). Controls for case Set 1 were a random sample of participants who had mild knee pain at study entry which was not severe at 18-month follow-up (characteristic pain intensity <70 out of 100). Controls for case Set 2 were a random sample of participants who had mild knee pain at 18 months which was not severe at 36 months. The second set of controls was selected after removal of the first set of controls (ie selection without replacement). Each set of cases and controls was mutually exclusive (ie no participant could appear in more than 1 group).

### Standardising Time

**Figure 1:**
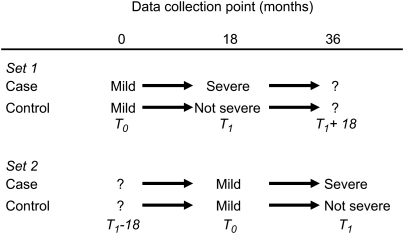
Selection and definition of cases and controls.

For the main analyses the 2 sets of cases were combined and compared to the combined control groups. T_0_ represented the time at which both cases and controls had mild pain. T_1_ was the time 18 months later when cases were reporting severe pain and controls were reporting nonsevere pain. We refer to the period between T_0_ and T_1_ hereafter as the “index period”. The selection of cases and controls and timing of measures is represented in Fig 1.

### Data Analysis

The sociodemographic and clinical-history characteristics of cases and controls were described. Between-group differences between cases and controls were calculated at the beginning and at the end of the index period. Results for dichotomous data were expressed as percentage differences with 95% confidence intervals (95%CI). For numerical data, ie number of pain areas, WOMAC-PF, HAD anxiety and depression scores, the mean and standard deviation at each time point was calculated and between-group differences expressed as mean differences with 95%CI. The 95%CI expresses the precision of the estimate of mean between-group difference obtained from the sample and is more informative than a simple p-value.

We conducted 2 additional separate subgroup analyses based on only 1 set of cases and controls. Set 1 (in which cases and controls were selected from baseline and 18-month data) provided information on pain experience 18 months after the index period. Set 2 (in which cases and controls were selected from 18- and 36-month data) provided information on pain experience 18 months before the index period.

## Results

After removing 16 participants with an existing diagnosis of inflammatory disease at study entry (n = 16), 433 participants were eligible for Set 1, having reported mild knee pain at study entry and provided pain-intensity data at 18-month follow-up. Of these, 28 (6.2%) had severe knee pain at follow-up and qualified as cases. To act as controls for these cases, 112 participants were randomly selected from the remaining 405 participants. For Set 2, 361 participants reported mild knee pain at 18-month follow-up and provided pain-intensity data at 36-month follow-up. Of these, 29 (7.8%) had severe knee pain at follow-up. From the remaining 332 participants, 116 were randomly selected to act as controls for this second set of cases.

Cases and controls were similar with respect to age, total time since onset of knee problem, and previous knee surgery (Table 1). There was a higher proportion of females among cases compared with controls, although this difference was statistically nonsignificant (difference 10.5%; 95%CI: –3.9, 23.6).

**Figure 2:**
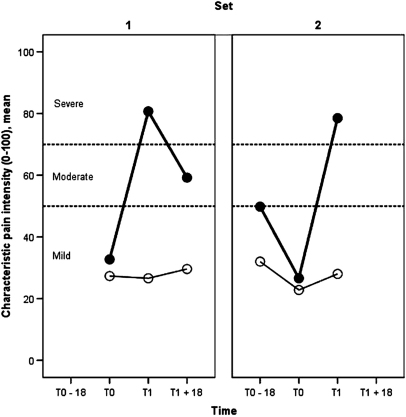
Mean characteristic knee-pain intensity over time for each set of cases (closed marker) and controls (open marker).

Fig 2 confirms the basis of selecting cases and controls: namely, substantial deterioration in knee-pain intensity over the 18-month index period among cases and comparable mild pain intensity at the start of the index period among controls.

### Comparison of Pain and Health Characteristics

Substantial deterioration in knee-pain intensity was associated with an increase in pain persistence, night pain, pain extent, functional limitation, and depressive symptoms during the index period (Table 2).

Although smaller, many of the between-group differences seen at the end of the index period (T_1_) were also apparent at the start of the index period (T_0_) when both cases and controls had mild characteristic-pain intensity (Table 2, column 2). Prior to substantial deterioration, cases were already more likely to report persistent knee pain, limited physical function, and depressive symptoms.

By contrast, anxiety symptoms and self-rated general health appeared to improve among cases, such that any initial differences between cases and controls were lost at the end of the index period. On further inspection of the data for both sets of cases and controls separately, it was clear that this was due to a strong secular pattern of change in the cohort as a whole with gradual improvements in average levels of anxiety and self-rated health seen from initial entry into the cohort onwards.

### Comparison of Coping and Appraisal

Substantial deterioration in knee pain was associated with an increase in catastrophic appraisals, praying and hoping, and use of coping self-statements (between-group differences for the latter reached borderline statistical significance at the end of the index period) (Table 3).

Between-group differences in catastrophising, and praying and hoping, were present at the start of the index period although, owing to the noncollection of CSQ data at cohort-study entry, this is based on 1 set of cases and controls only and was statistically nonsignificant.

There were no between-group differences either at the start or the end of the index period in the reported use of distraction, reinterpreting pain sensation, ignoring pain, or increased behavioral activities.

Overall, the presence of any of the 7 coping strategies was similar at the start of the index period but was significantly higher in cases compared to controls at the end of the index period.

### Comparison of Self-Treatment and Health Care Use

Substantial deterioration in knee pain was associated with a marked increase in the use of oral and topical analgesia, particularly oral opioids (Table 4). There was a modest increase in the use of specific knee exercises among cases although the absolute proportion of cases and controls doing specific knee exercises remained low. Dieting to lose weight showed a similar secular pattern to anxiety and self-rated health, becoming less common over time for controls and particularly for cases.

Forty-six percent of cases had a record of visiting their GP about their knee problem during the time when substantial deterioration had taken place. Only 1 in 5 controls had visited their GP. This difference between cases and controls persisted after the index period. In total, 82% of cases had a record of consulting their GP about their knee problem either during the index period or in the 18-month period following substantial deterioration. This compares with 24% of controls.

Between-group differences were apparent at the start of the index period (T_0_) (Table 4, column 2) although generally much less marked than the differences at the end of the index period (Table 4, column 3). Prior to deterioration, cases were more likely than controls to be using some form of oral or topical analgesia.

### Subgroup Analyses

The shaded columns in Tables 2 to 4 provide some possible insights into pain and health characteristics and treatment use before and after the index period, but are based on only 1 set of cases and controls and therefore need to be cautiously interpreted. Cases appeared to have higher characteristic pain intensity than controls both before and after the index period. This suggests that although cases and controls had similar levels of characteristic pain intensity at the beginning of the index period, individuals who went on to experience a substantial deterioration of their knee pain already had a different pain history from controls and, following substantial deterioration, did not, on average, return to the same level as controls.

## Discussion

### Summary of Main Findings

Substantial deterioration of knee pain, defined here as the transition from mild to severe characteristic knee pain over an 18-month period, is relatively uncommon in the general population. However, it has major implications for those affected. Substantial deterioration of knee pain is accompanied by an increase in the perceived frequency of knee pain, the presence of severe night pain, greater pain extent, increased limitation of daily activities, more depressive symptoms, and an increase in catastrophic thoughts, praying and hoping, and coping self-statements. Allowing for a degree of underrecording of knee-related general practice consultations,^23^ it seems likely that people experiencing substantial deterioration of knee pain do present their problem to the general practitioner, but there can be delays in doing so. The observed pattern of increased use of oral and topical analgesia, and, to a lesser extent, specific knee exercises reflects the combined provision of primary health-care and self-care efforts (eg opioid analgesia are prescription-only medications but paracetamol and oral NSAIDs could be obtained over the counter).

Substantial deterioration does not generally occur from a “standing start”. Cases and controls were selected on the basis of having the same level of characteristic knee pain intensity at the start of the index period. They also had a similar age and total time since problem onset. However, prior to deterioration, cases already clearly differed from controls on a number of other measures (eg pain frequency, functional limitation), had significantly higher pain intensity in the 18 months prior to the index period, and were already engaged in more efforts to control their pain (eg opioid intake). Together these findings suggest that substantial worsening may often be 1 phase in a history of fluctuating pain. Mild characteristic pain for controls may represent the true underlying severity of their problem: For cases, it may indicate a period where pain, usually more severe, was successfully, but temporarily, under control. Substantial deterioration may represent the loss of effective pain control.

### Comparison with Previous Literature

The proportion of individuals reporting substantial worsening was consistent with previous surveys of chronic-pain populations using similar repeated measures of pain intensity.^13,60^ Nevertheless, the true incidence of significant deterioration will be underestimated in our study due to flare-ups that resolved before remeasurement 18 months later. Unobserved flare-ups among controls would tend to result in conservative estimates of the differences between cases and controls at the end of the index period.

Our findings extend previous cross-sectional studies that have described differences in coping and treatments between individuals with mild and severe pain by demonstrating that the transition from mild to severe knee pain is accompanied by certain changes in coping and treatment within those affected. Catastrophising was not the focus of our study but our findings appear to confirm its importance in older adults with knee pain and OA^12^ and are broadly consistent with the notion that pain-related catastrophising is both dispositional and situation-influenced.^49,52^ Our results complement those from therapeutic trials of coping-skills training. Such trials have demonstrated the ability of such interventions in clinical settings to reduce patients' reliance on passive coping techniques with a favorable impact on perceived pain intensity and impact on daily activities.^24,25^ Our study shows how a substantial worsening of pain is accompanied by the “activation” of these passive coping strategies.

Our findings that neither anxiety nor self-rated health appeared to be affected by a substantial deterioration in knee-pain severity were unexpected. There is strong evidence for a cross-sectional association between anxiety disorders and arthritis and other painful conditions,^9,17,46^ and between frequency of chronic pain and poor self-rated health.^31^ We anticipated that substantial deterioration in knee pain would result in an increase in anxiety symptoms and poorer self-rated health. Instead, a secular trend in the direction of lower anxiety and better self-rated health over time was observed within all cohort members but was particularly marked in cases (who generally began with higher anxiety and poorer self-rated health than controls). Although attrition was low, the observed trends for anxiety and self-rated health are due to selective loss to follow-up. Participants with high levels of anxiety or poor self-rated health were more likely to be lost at the next follow-up (data not shown). Differences between cases and controls on these 2 features at the end of the index period are therefore likely to be underestimated in our study.

### Strengths and Limitations

There is continuing interest in defining, describing, and explaining clinical important change. Most previous research focuses on improvement following therapeutic intervention.^11^ Yet given the large numbers of individuals in the general population with relatively mild pain, whose only concern about it may be the prospect of it significantly worsening, there is a need also to investigate the other side of the coin; significant deterioration. Our study provides original descriptive data on this using a justifiable (but relatively stringent) definition based on commonly-used measures of pain intensity. Cases and controls were sampled from a well-characterised cohort with low attrition and using prospectively-gathered repeated measures. Despite these strengths, there are several limitations of the present study that deserve mention.

The number of cases was relatively small and our findings, particularly those from the subgroup analyses, should be interpreted with caution and regarded principally as exploratory rather than hypothesis-generating. For this reason, we have confined ourselves to calculating unadjusted mean between-group differences and an estimate of the precision of these rather than p values. If we accept that the transition from mild to severe pain is an important one to investigate, our study suggests that much larger sample sizes would be required given the relative infrequency of this large change in symptoms in members of the general population. Caution is needed also given the number of comparisons made in the present analyses raising the prospect of Type I errors and the fact that our unadjusted analyses could not control for any baseline differences.

One disadvantage of attempting to cover a very broad range of domains within the same study was the need to use brief questionnaires or single items for some domains to minimize respondent burden. Coping and appraisal, for example, was based on the 1-item CSQ, with items dichotomized. Such data reduction was felt to be necessary given the highly-skewed distribution although the effect of this is that increases or decreases in the frequency of use of different coping strategies could not be directly quantified. The 2-item CSQ has recently been validated in older adults^50^ but misclassification will be higher for the 1-item version. Several recommended treatments were not included in our study (eg aids and adaptations, supports/braces, general aerobic exercise) or the source (eg prescribed vs OTC) not specified. Future studies would benefit from extended measurement of catastrophising and treatment approaches mapping to all those recommended in current guidelines, although this should be tempered by the expectation that recommended measures often change within the time scale needed for a study of this kind.

The precise temporal sequence of changes in pain intensity and changes in coping observed in this study cannot be fully disentangled as our primary analyses are based on 2 time points. We have referred to many of the changes accompanying substantial deterioration in pain intensity as “effects”. This assumption of temporal sequence is reasonable for treatment use (eg increased use of oral analgesia is an effect of substantial worsening of knee pain rather than the other way round) but may not be true of other observed changes (eg worsening pain may be an effect of increased catastrophising).

Our study used between-group differences to represent the “average effects” of a substantial worsening in characteristic knee-pain intensity. Average effects can still mask differences between individuals in how they respond to substantial worsening of pain. Our study indicates what tends to happen when mild knee pain becomes severe. Idiographic within-person daily process studies have investigated sequential day-to-day relationships between pain, mood, and coping at the level of individuals,^1,26,53^ and the value of such studies has been persuasively argued.^2,51^ Our approach enabled us to investigate changes in characteristic rather than current pain. We were able to define a specific transition a priori and applied a stringent and reproducible definition of change corresponding to a 50% increase in intensity. It is unclear whether the processes described in daily diary studies could be generalized to these conditions. While an idiographic approach could still be adopted for these longer-term and extreme transitions, several years of follow-up would be required before sufficient numbers of repeated measures are obtained and sufficient cases accrued. We are continuing follow-ups at 18-month intervals of this and other similar cohorts, and hope that this effort will provide an opportunity to do more follow-up in the future.

### Implications for Clinical Practice and Future Research

Our study is descriptive but serves to illustrate the process of substantial deterioration that may precede presentation to the general practitioner. Low mood, catastrophising, and praying and hoping for the pain to go away may be important features of the presenting problem in primary care and greater efforts are needed in translating principles of cognitive-behavioral approaches into practical interventions in this setting. Understanding why some individuals experience relatively rapid and significant deterioration in pain is a priority for future research, although the notion of a loss of adequate pain control must be considered alongside the search for distinct triggering events.

Improving the uptake of core nonpharmacological treatments is a priority for clinical practice. Current guidelines and recommendations emphasize the importance of weight loss and exercise therapy for all patients with knee OA.^33,63^ Our study confirms recent study findings of a relatively low use of these core treatments and that these treatments are often “stepped over” when knee pain significantly worsens, in favor of adjunctive pharmacological treatment.^39^ One hypothesis for future research arising from our work is that individuals suffering recurrent major fluctuations in pain may become locked in a cycle of “crisis management” where beneficial long-term changes in lifestyle (weight control, physical activity and exercise) are interrupted or postponed. As a relatively small minority of older adults experience the sort of substantial deterioration in knee pain identified in our study, it would appear to be ideally suited to qualitative research methods which would complement the present study by providing a more detailed account of the process of substantial deterioration in knee pain and peoples' responses to it. Indeed, the findings of 1 such previous qualitative interview study showing the “take as needed” attitude to pain medications adopted by people with OA^44^ provides an excellent illustration of the value of complementing quantitative and qualitative research methods in this field. Applying such mixed methodological approaches would be 1 line for future research in this topic area.

## Figures and Tables

**Table 1:** Descriptive Characteristics of Cases and Controls at Study Entry

	Cases (n = 57)	Controls (n = 228)
Age (years) at study entry, mean (SD)	66.3 (9.2)	64.6 (8.2)
Female gender, n (%)	36 (63)	120 (53)
Time since onset of current knee problem, n (%)		
<1 year	9 (16)	36 (16)
1 to <5 years	20 (35)	76 (33)
5 to <10 years	11 (19)	51 (22)
10+ years	17 (30)	65 (29)
Previous knee surgery, n (%)	6 (11)	20 (9)

**Table 2:** Differences Between Cases and Controls at Each Time Point: Pain and Health

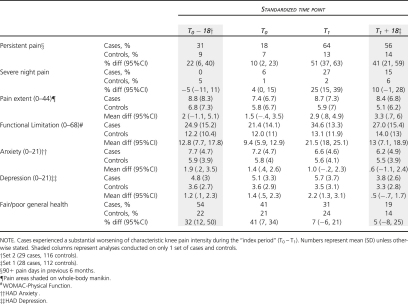

**Table 3:** Differences Between Cases and Controls at Each Time Point: Coping and Appraisal

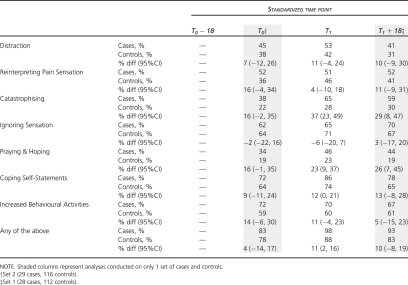

**Table 4:** Differences Between Cases and Controls at Each Time Point: Treatment^∗^